# Recent developments in diagnosis and risk stratification of non-ST-elevation acute coronary syndrome

**DOI:** 10.1007/s12471-020-01457-3

**Published:** 2020-08-11

**Authors:** G. W. A. Aarts, J. Q. Mol, C. Camaro, J. Lemkes, N. van Royen, P. Damman

**Affiliations:** 1 Department of Cardiology, Radboud University Medical Centre, Nijmegen, The Netherlands; 2 Department of Cardiology, Amsterdam UMC, location VUMC, University of Amsterdam, Amsterdam, The Netherlands

**Keywords:** NSTE-ACS, Intracoronary imaging, Troponin

## Abstract

In the past year, a number of important papers have been published on non-ST-elevation acute coronary syndrome, highlighting progress in clinical care. The current review focuses on early diagnosis and risk stratification using biomarkers and advances in intracoronary imaging.

## Dutch contribution to the field

- PECTUS trial on intracoronary imaging of vulnerable plaque.
- ARTICA trial on prehospital triage.

## Introduction

In addition to some universal clinical markers of risk, such as advanced age, diabetes and renal insufficiency, the initial clinical presentation is highly predictive of early prognosis in patients with non-ST-elevation acute coronary syndrome (NSTE-ACS). Guidelines recommend the quantitative assessment of ischaemic risk by means of risk scores such as the GRACE score, which is superior to clinical assessment alone. The GRACE risk score includes age, systolic blood pressure, pulse rate, serum creatinine, Killip class at presentation, cardiac arrest at admission, elevated cardiac biomarkers and ST deviation on the electrocardiogram. A number of important papers have been published in the past year highlighting progress in early diagnosis and risk stratification. The current review focuses on the use of biomarkers and intracoronary imaging. As a celebration of the European Society of Cardiology 2020 congress we will discuss important upcoming or published Dutch studies related to these subjects.

## Biomarkers in diagnosis and risk stratification of NSTE-ACS

In patients with chest pain, one of the main diagnostic concerns is whether the chest pain is due to an acute coronary syndrome (ACS). In suspected ACS, the diagnostic foundation is a combination of a 12-lead electrocardiogram (ECG), clinical evaluation and cardiac troponin measurements [1]. After obtaining an ECG, the diagnosis is relatively straightforward in patients with an acute ST-segment-elevation myocardial infarction (STEMI). However, when ST-segment elevations are absent on the ECG, further evaluation is required in order to rule in or rule out an NSTE-ACS. Although only 10–20% of chest pain patients have an ACS, chest pain is one of the main complaints in the emergency department (ED), accounting for up to 10% of all ED visits [2–7]. Moreover, ED overcrowding, which is associated with worse patient outcomes, is a growing phenomenon [8, 9]. Therefore, early risk stratification and a shorter time to diagnosis in chest pain patients is important.

### 0 h/1 h algorithm

Measuring cardiac troponin with sensitive or high-sensitivity cardiac troponin (hs-cTn) assays to rule in or rule out NSTE-ACS is recommended by the European Society of Cardiology (ESC) [1]. If hs-cTn assays are available, using a 0 h/1 h algorithm as an alternative to the 0 h/3 h algorithm is also recommended. Using a 1-hour algorithm may reduce the delay to diagnosis, leading to shorter stays in the ED, less overcrowding and lower costs [10–15]. The applicability and validity has recently been investigated in several studies.

Boeddinghaus et al. evaluated the validity of the 0 h/1 h algorithm by prospectively enrolling patients presenting to the ED with symptoms suggestive of acute myocardial infarction in three diagnostic studies according to age (<55 years, ≥55 to <70 years, and ≥70 years). Hs-cTn T and hs-cTn I plasma concentrations were measured at presentation and after 1 h. In all age groups, the rule-out safety was very high, with a sensitivity of >99.3%.[16] However, with increasing age, the overall specificity and the accuracy of rule-in significantly decreased. Therefore, Boeddinghaus et al. suggest that alternative slightly higher cut-off concentrations may be considered for older patients. The authors confirmed their findings in two validation cohorts. However, when age is incorporated in the algorithm in order to improve the specificity, adding other confounders such as chronic kidney disease, chronic heart failure and atrial fibrillation should also be considered [17]. Twerenbold et al. evaluated the real-world performance of the 0 h/1 h algorithm and confirmed its excellent applicability [18]. The routine use of the 0 h/1 h algorithm resulted in very low 30-day major adverse cardiac event (MACE) rates in the rule-out group and in outpatients (0.2% and 0.1%, respectively). Moreover, routine use of the 0 h/1 h algorithm resulted in an average short ED stay of 2.5 h, suggesting that implementation of the 0 h/1 h algorithm might help to prevent ED overcrowding. Chew et al. evaluated the 0 h/1 h algorithm in a randomised setting in the RAPID-TnT trial.[19] In this multicentre trial, patients with suspected ACS were randomised to either the 0 h/1 h hs-cTn algorithm or the 0 h/3 h standard algorithm in which the troponin T assay’s high-sensitivity performance characteristics were masked. Patients randomised to the 0 h/1 h arm were more likely to be discharged from the ED, had a shorter length of stay in the ED and underwent less functional cardiac testing. The negative predictive value of the 0 h/1 h algorithm was 99.6% for MACE.

To summarise, several studies have shown that the 0 h/1 h algorithm recommended by the ESC is an excellent fast rule-in and rule-out tool enabling more rapid discharge and less additional testing in suspected ACS patients.

### Pre-hospital rule out

Ruling out ACS in the pre-hospital setting could have major medical and economic value. Schols et al. conducted a nationwide flash-mob study in the Netherlands to evaluate the safety of the Marburg Heart Score (MHS) to rule out ACS [20]. The MHS is a clinical decision rule based on five signs and symptoms, which was designed to identify patients with a low probability of ACS as the underlying cause of chest pain in the primary care population [21, 22]. Although ruling out ACS in the primary care setting without additional tests, such as the ECG and cardiac troponin measurements, sounds attractive, Schols et al. showed that ACS could not be safely ruled out using the MHS. The FamouS Triage study group has shown that it is possible to identify low-risk patients in the pre-hospital setting by assessing the modified History, Electrocardiogram, Age, Risk factors and Troponin (HEART) score [23, 24]. Moreover, they have shown that incorporating a point-of-care (POC) troponin T test into the HEART score enables ambulance paramedics to assess the complete HEART score and rule out ACS in low-risk patients at home [25]. In order to further evaluate the possibilities of ruling out ACS at home, the ARTICA (*A*cute *R*ule out of non‑S*T *elevation acute coronary syndrome in the pre-hospital setting by HEART score assessment and a single po*I*nt-of-*C*Are troponin) trial is currently being conducted [26]. The ARTICA trial is a randomised trial with a primary objective to evaluate the cost-effectiveness of ruling out ACS at home. Low-risk patients are identified by assessment of the H, E, A and R components of the HEART score and then randomised to either transfer to the ED (standard care) versus POC troponin T measurement at home. If the POC troponin T value is below the limit of detection (40 ng/l), an ACS is considered ruled out and the care of the patient is transferred to the general practitioner.

## Intracoronary optical coherence tomography for diagnosis and risk stratification

Intracoronary imaging, using intravascular ultrasound (IVUS) or optical coherence tomography (OCT) has become widely implemented in the cathlab. Recently a consensus document for the clinical use of intracoronary imaging in ACS was published, highlighting several indications for OCT [27]. One of the key roles for OCT in ACS is its ability to assist in identifying a culprit lesion, based on the presence of thrombus. In the absence of significant coronary artery disease (CAD), OCT may assist in the evaluation of non-atherosclerotic aetiologies. In addition to determining the cause of an ACS, OCT enables in vivo assessment of plaque composition. Determining the morphological make-up of atherosclerotic lesions might help with risk stratification by identifying high-risk plaques or patients.

### OCT-derived vulnerable plaque features

Several characteristics of morphological lesions have been associated with increased vulnerability to plaque rupture in retrospective studies [28]. In general these vulnerable lesions have a large necrotic core with a thin, inflamed overlying fibrous cap, and are referred to as thin-cap fibroatheromas. In the prospective observational CLIMA study, OCT-derived vulnerable plaque features of lesions in the left anterior descending artery were correlated with a composite endpoint of cardiac death and target segment myocardial infarction at 12-month follow-up [29]. It was shown that lesions with the combination of four high-risk features (a minimal lumen area of <3.5 mm^2^, a fibrous cap thickness of <75 µm, a lipid arc of >180°, and macrophage infiltration) were associated with the endpoint with a hazard ratio of 7.54. CLIMA was the first OCT study to prospectively link plaque vulnerability to clinical endpoints. However, because OCT imaging in this study was performed on a single predefined segment, the question remains whether ‘targeted’ OCT imaging of angiographic lesions can improve risk stratification, and potentially guide treatment decisions. This gap in knowledge is currently being addressed by the COMBINE (NCT02989740), and PECTUS-obs study (NCT03857971).

### Vulnerable plaque treatment

The ability to identify vulnerable coronary lesions might have therapeutic consequences. In addition, intracoronary imaging could be used to quantify vulnerability of the complete epicardial coronary system, thereby identifying ‘vulnerable patients’. Aggressive systemic therapy might be beneficial for vulnerable plaques or patients. In addition, vulnerable plaques might be a target for focal therapy by means of percutaneous coronary intervention. Both systemic and local therapies are the subject of multiple active studies. For instance, The HUYGENS (NCT03570697) and PACMAN-AMI (NCT03067844) trials aim to see if treatment with PCSK9 inhibition results in stabilisation of plaque morphology and fewer clinical events, whereas the PROSPECT-ABSORB (NCT02171065) and PREVENT (NCT02316886) studies focus on preventive stenting of vulnerable plaques.

### Healed plaques

Healed plaques are atherosclerotic lesions with a layered OCT appearance. This phenotype is believed to be the result of repair processes of ruptured or eroded plaques [30]. Identification of these lesions with OCT has recently been validated against histology and has gained considerable attention in the last year [31]. In an observational cohort study of 105 patients who had undergone coronary angiography with OCT imaging of non-culprit lesions, patients were divided into two groups based on the two extremes of the clinical spectrum of CAD [32]. The first group contained patients who had long-standing stable CAD, and the second group contained patients with a history of multiple recurrent ACS. In the long-term stable CAD group, healed plaques were seen in about one-third of patients, whereas in the recurrent ACS group hardly any healed plaques were observed. The authors argued that plaque healing takes place after plaque rupture or erosion if thrombosis-resisting factors prevail, whereas a clinically manifest ACS occurs in a more pro-thrombotic environment, and that detection of healed plaques could therefore help identify patients who are relatively protected from developing acute occlusive thrombosis. A different OCT study of pre-intervention ACS culprit lesions showed a layered phenotype in 29% of lesions [33]. In contrast to the previous study, they found that patients with healed culprit plaques were more often associated with a history of myocardial infarction. Additionally, plaque rupture, lipid plaques, and thin-cap fibroatheromas were more prevalent in healed culprit plaques and the prevalence of healed plaques increased with an increasing degree of percent area stenosis. The authors therefore concluded that the associations with plaque vulnerability may outweigh the protective mechanism of plaque healing and might actually predispose patients to future acute coronary events. In a following study of the same cohort of ACS patients, it was shown that this association with vulnerable plaque characteristics also extended to healed non-culprit lesions [34].

## Future perspective

We have discussed recent developments in the diagnosis and risk stratification of NSTE-ACS. With regards to the early diagnosis, multiple reports have shown the diagnostic accuracy of the 0 h/1 h algorithm enabling earlier ruling in and ruling out of NSTEMI. We expect improvements in the POC assays, resulting in an even higher accuracy in the pre-hospital triage in the ambulance or even by the general practitioner. NSTE-ACS is associated with long-term recurrent ischaemic events. The concept of identifying vulnerable plaques or patients based on the characteristics of morphological lesions seems promising, and might further improve risk stratification if added to existing risk models. However, more prospective studies with clinical outcome data are needed to validate this strategy.
